# Do physician communication skills influence screening mammography utilization?

**DOI:** 10.1186/1472-6963-12-219

**Published:** 2012-07-25

**Authors:** Ari-Nareg Meguerditchian, Dale Dauphinee, Nadyne Girard, Tewodros Eguale, Kristen Riedel, André Jacques, Sarkis Meterissian, David L Buckeridge, Michal Abrahamowicz, Robyn Tamblyn

**Affiliations:** 1 Clinical and Health Informatics Research Group, McGill University, 1140 Pine Avenue West, Montreal, QC, H3A 1A3, Canada; 2 Quebec College of Physicians, 2170 René-Lévesque West Boulevard, Montreal, QC, H3H 2T8, Canada; 3 Cedars Breast Centre, McGill University Health Centre, 687 Pine Avenue West, S10.22, Montreal, QC, H3A 1A1, Canada; 4 Department of Epidemiology and Biostatistics, McGill University, 1020 Pine Avenue West, Montreal, QC, H3A 1A2, Canada

## Abstract

**Background:**

The quality of physician communication skills influences health-related decisions, including use of cancer screening tests. We assessed whether patient-physician communication examination scores in a national, standardized clinical skills examination predicted future use of screening mammography (SM).

**Methods:**

Cohort study of 413 physicians taking the Medical Council of Canada clinical skills examination between 1993 and 1996, with follow up until 2006. Administrative claims for SM performed within 12 months of a comprehensive health maintenance visit for women 50–69 years old were reviewed. Multivariable regression was used to estimate the relationship between physician communication skills exam score and patients’ SM use while controlling for other factors.

**Results:**

Overall, 33.8 % of 96,708 eligible women who visited study physicians between 1993 and 2006 had an SM in the 12 months following an index visit. Patient-related factors associated with increased SM use included higher income, non-urban residence, low Charlson co-morbidity index, prior benign breast biopsy and an interval >12 months since the previous mammogram. Physician-related factors associated with increased use of SM included female sex, surgical specialty, and higher communication skills score. After adjusting for physician and patient-related factors, the odds of SM increased by 24 % for 2SD increase in communication score (OR: 1.24, 95 % CI: 1.11 - 1.38). This impact was even greater in urban areas (OR 1.30, 95 % CI: 1.16, 1.46) and did not vary with practice experience (interaction p-value 0.74).

**Conclusion:**

Physicians with better communication skills documented by a standardized licensing examination were more successful at obtaining SM for their patients.

## Background

Screening mammography (SM) is a proven strategy for reducing breast cancer mortality [1,2]. Despite vigorous public education campaigns, funded screening programs, and multiple national / international practice guidelines [3,4], 30 to 40 % of women do not take advantage of this low cost, high impact test [5]. Patient and health care system-related issues may partially explain SM underutilization [5-9]. Physician-related factors, such as age, gender, years since graduation, practice setting, patient population profile and adherence to maintenance-of-competence activities can also affect SM use [5,8,10-13]. Physician recommendation is another important predictor that women will undergo a SM, as discussions in the medical setting have been shown to significantly affect patient decisions [14,15]. However, there is a significant gap in the scientific understanding of the physician-patient communication process, particularly in regard to decisions concerning potentially life-saving screening tests [16].

Although viewed more as the “art” of medicine, communication skills constitute an ensemble of items that refer to specific, attainable, and measurable objectives defining a clinical encounter [17-20]. These skills impact patient satisfaction, adherence to treatment plan, clinical outcomes and malpractice litigation [21-24]. While intuitively suspected, the relationship between physician communication skills and SM use has not yet been evaluated, due to lack of data on communication skills assessment and service utilization linked to the same physician. The purpose of this study is to assess the association between physicians’ communication skills and the use of SM services in a population of female patients aged 50 to 69.

## Methods

### Context

This study was conducted in Quebec (Canada). The province maintains administrative databases of insured persons and claims for all medical services remunerated on a fee-for-service basis. Claims include information on diagnosis, procedure, date, location, referring physician and physician and patient identifiers. Under universal health coverage, 99 % of residents have provincial health insurance, and >95 % of services are remunerated on a fee-for-service basis.

Since 1992, to be licensed in Canada, all physicians must complete the Medical Council of Canada (MCC) national clinical skills examination (MCCQE2) after the traditional written examination assessing knowledge and clinical decision-making (MCCQE1). The MCCQE2 assesses communication, history and physical examination skills and clinical management through direct observation of performance in 18–20 standardized patient cases.

### Design

Appropriate ethical / legal clearances were obtained from the MCC, Quebec Privacy Commission, the provincial health insurance agency (RAMQ), and McGill University institutional review board.

We assembled a prospective cohort of physicians who completed the MCCQE2 between 1993 and 1996, and provided breast health care in Quebec (family/general physicians, gynecologists, general surgeons). The MCC and Quebec medical regulatory authority assembled the physician cohort, replacing nominal data with a study number. RAMQ retrieved and anonymized all claims for the 5.8 million patients seen by study physicians between 1993 and 2007.

Women aged 50–69 years, cared for by study physicians between 1993 and 2006, and eligible for SM were identified. To ensure these patients were part of the study physician’s practice and that the physician had the opportunity to discuss breast cancer screening, only women who had a health maintenance visit with complete physical exam by the study physician in the year prior to the visit were included. Only the first eligible visit (index visit) was selected to avoid potential biases related to selective retention of more adherent patients. Women with suspected or confirmed breast cancer, defined as a claim for lumpectomy, mastectomy, modified radical mastectomy, or a breast cancer diagnosis prior to their visit were excluded. The resulting cohort was followed for 12 months post index visit to determine SM receipt.

### Measurement

***Screening mammography*** was defined as a claim for a bilateral two-view mammogram within 12 months of the index visit. Service codes for SM are different from those for diagnostic mammography.

***Physician communication skills*** were assessed by the MCCQE2 [25,26]. *Doctor-patient communication skills* are assessed by trained observers using case-specific checklists. They grade examinees on 3–4 cases selected to represent challenging situations where effective communication is required (e.g. refusal of treatment for a terminal illness, contraception counseling). *Data acquisition skills* are assessed in 5–10 minute interactions with 16–17 cases representing common important problems in practice (e.g. history and physical examination for a patient with abdominal pain). The reliability of MCCQE2 examination sub-scores in different administrations was 0.25-0.50 for the communication sub-score and 0.59-0.75 for data acquisition (cronbach alpha) [27]. The communication sub-score has been shown to have excellent predictive validity for many outcomes (appropriate antibiotic use [28], persistence to anti-hypertensive medication [29], complaints to regulatory authorities [30]). Scores are standardized to a mean of 500 (SD 100), based on scores for first-time takers from Canadian medical schools.

#### Physician-related potential confounders

Sex, specialty, international medical graduate status were supplied by the medical regulatory authority. We used the MCCQE1 score to adjust for differences in medical knowledge and clinical decision-making ability [31]. Practice experience may attenuate associations between communication and SM use. Years in practice were calculated based on number of months where the physician billed one or more medical services [32].

#### Patient-related potential confounders

Age at index visit was calculated using birth date supplied by the RAMQ. Education and household income were measured by linking the patient’s postal code to 1996 census information on the proportion with a high school degree and mean family income in that neighborhood (approximately 366 households per postal code). Postal code was used to classify location as rural or urban, based on Statistics Canada definitions [33]. Co-morbidities were measured using the Charlson Comorbidity Index, using International Classification of Diseases 9 (ICD-9) codes in claims in the year before the index visit. Hospitalization in the year before the index visit was determined using the establishment code (private clinic, emergency room, hospital, etc.) on claims. Two indicators of patient breast-related health care in the year prior to the index visit were measured as characteristics that may modify the likelihood of SM: 1) a breast biopsy, 2) a bilateral mammogram. A dichotomous indicator was used to adjust for the availability of a province-wide screening program during follow-up (started in 1998) as it had the effect of increasing diagnostic mammography [34,35].

### Analysis

We tested the hypothesis that physician communication skills are associated with the probability of a woman receiving SM using the generalized estimating equation (GEE) extension of multivariable logistic regression (SAS Version 9.2). An exchangeable covariance structure was used to account for clustering of patients within physicians. The patient was the unit of analysis and the occurrence of SM during the 12-month follow-up was the binary outcome. Models were adjusted for physician and patient characteristics. To assess whether the association between communication skills and mammography varied by patient urban/rural status or by physician experience we tested the interaction between skill scores and these two variables in separate multivariable GEE models.

## Results

Among 6,667 physicians who took the MCCQE2 examination between 1993 and 1996, 1,116 entered practice in Quebec, and 413 entered specialties traditionally providing breast health care. Earliest practice entry date was 1994, latest was 2005. Between 1994 and 2006, 96,708 eligible women made 690,551 visits to study physicians, from which we selected the index visit. The mean years in practice during the follow-up was 3.6 years (SD: 2.7) (Table 1). Gynecologists provided care for <2 % of our study population; therefore they were regrouped with general surgeons.

**Table 1:** Demographic, training and practice characteristics of the 413 study physicians

**Physician Characteristics**	**N = 413**
**Demographics**	**No. (%)**
**Sex**	
Female	269 (65.1)
Male	144 (34.9)
**Age at MCCQE2 exam date**	
<25 years old	141(34.1)
25-35 yrs old	248 (60.0)
≥35 years old	24 (5.8)
**Medical School**	
Canadian	389 (94.2)
International graduate	24 (5.8)
**Specialty**	
Family medicine / general practice	371 (89.8)
Medical specialty	10 (2.4)
Surgical specialty	32 (7.7)
**Medical Knowledge & Clinical Decision Making Ability**	**mean (SD)**
**MCCQE1 Written Examination Overall Score**	511.3 (76.2) IQR = 103
**Communication Ability**	**mean (SD)**
**MCCQE2 Clinical Skills Examination Overall Score**	526.9 (84.8)^a^
Communication sub-score	510.6 (97.0)^a^
Data acquisition sub-score	531.1 (92.5)^a^
**Practice Experience**	**mean (SD)**
**Number of years of practice during follow-up**	3.6 (2.7)

Overall, 65 % of study physicians were female, most trained in family medicine at a Canadian medical school, and were 25–34 years old when taking the MCCQE2 examination (Table 1). The mean overall score on MCCQE2 was 526.9, and 511.3 for MCCQE1 (average for first-time examination takers from Canadian medical schools was 500). The mean scores for the communication and data acquisition sections were 510.6 and 531.1 respectively; however the range for both scores was 6–7 standard deviations wide.

The mean age of eligible women seen by study physicians was 57.2 years (SD: 5.77), mean family income in their postal code area was $51,063, and 88.2 % lived in urban centers (Table 2). Most women had no significant co-morbidity, and only 8.5 % had more than one hospitalization in the year prior to their first visit with the study physician. Few patients had breast health-related procedures (Table 2). Only 27.5 % had a SM in the past year, and 51.6 % of visits occurred after the provincial screening program was implemented.

**Table 2:** Patient characteristics in the twelve months following the visit to the study physician

**Patient Characteristics**	**N = 96 708**
**Demographics**	**mean (SD)**
**Age**, in years	57.2 (5.8)
**Percent with high school diploma**	31.8 (13.0)
**Family income** (*in Canadian dollars)*	51 063 (23 116)
**Patient region**	**No. (%)**
Rural	11 410 (11.8)
Urban	85 298 (88.2)
**Health Status**	**No. (%)**
**Charlson Comorbidity Index**	
0	73 507 (76.0)
1-2	20 896 (21.6)
3-4	1 530 (1.6)
≥5	775 (0.8)
**Hospitalization in past year**	
No hospitalization	79 879 (82.6)
1 hospitalization	8 600 (8.9)
≥ 2 hospitalizations	8 229 (8.5)
**Breast Related Health Care**	**No. (%)**
**Breast biopsy in past year**	
No biopsy	95 918 (99.2)
Biopsy	790 (0.8)
**Mammogram in past year**	
No	70 089 (72.5)
Yes	26 619 (27.5)
**Provincial self-referral screening program available**	
No	46 841 (48.4)
Yes	49 867 (51.6)

Overall, 33.8 % had an SM in the 12 months after the index visit with the study physician (Table 3). After adjusting for physician and patient characteristics, the odds of having a mammogram increased with higher income, residence in a rural area and decreased with age and education. A Charlson co-morbidity index value ≥5 decreased the odds of having a SM by 16 % (OR: 0.84, 95 % CI: 0.72, 0.98). The odds of ordering an SM were 38 % greater if there was a prior breast biopsy negative for cancer (OR: 1.38; 95 % CI: 1.15, 1.65), and almost 4 times greater if there was no SM in the past year (OR: 3.94, 95 % CI: 3.47, 4.48).

**Table 3:** Patient characteristics and their relationship with receiving mammography screening in the 12 months after visiting a study physician

	**Mammography screening**			
**Patient Characteristics**	**Yes**	**No**	**Odds Ratio**^**a**^**(95% CI)**	**P- Value**
	**N=32 689**	**N=64 019**		
**Demographic**	**mean (SD)**	**mean (SD)**		
**Age**, per 10 year increase	56.9 (5.6)	57.4 (5.9)	0.99 (0.98-0.99)	<0.001
**Percent high school drop-outs**, per 20% increase	31.3 (12.9)	32.1 (13.1)	0.94 (0.90-0.98)	0.001
**Family income**, in dollars, per $25,000 increase^b^	52,302 (23,302)	50,431 (22,754)	1.08 (1.06-1.11)	<0.001
**Patient region**	**No. (%)**	**No. (%)**		
Urban	28 263 (86.5)	57 035 (89.1)	reference	
Rural region	4 426 (13.5)	6 984 (10.9)	1.44 (1.23-1.69)	<0.001
**Health Status**	**No. (%)**	**No. (%)**		
**Charlson Comorbidity Index**				
0	25 595 (78.3)	47 912 (74.8)	reference	
1-2	6 448 (19.7)	14 448 (22.6)	0.88 (0.84-0.91)	<0.001
3-4	416 (1.3)	1 114 (1.7)	0.71 (0.63-0.82)	<0.001
>=5	230 (0.7)	545 (0.9)	0.84 (0.72-0.98)	0.02
**Hospitalization in past year**				
No hospitalization	27 642 (84.6)	52 237 (81.6)	reference	
≥ 1 hospitalization	5 047 (15.4)	11 782 (18.4)	0.97 (0.93-1.02)	0.21
**Breast Related Health Care**	**No. (%)**	**No. (%)**		
**Breast biopsy in past year**				
No biopsy	32 404 (99.1)	63 514 (99.2)	reference	
Biopsy	285 (0.9)	505 (0.8)	1.38 (1.15-1.65)	<0.001
**Mammogram in past year**				
Yes	4 329 (13.2)	22 290 (34.8)	reference	
No (due for mammogram)	28 360 (86.8)	41 729 (65.2)	3.94 (3.47-4.48)	<0.001
**Self-referral screening available**				
No	17 945 (54.9)	31 922 (49.9)	reference	
Yes	14 744 (45.1)	32 097 (50.1)	0.86 (0.82-0.89)	<0.001

^a^Estimated using the generalized estimating equation (GEE) extension of multivariate logistic regression, adjusting for physician sex, specialty, medical school within Canada, years of practice, MCCQE1 exam score, MCCQE2 communication sub-score, MCCQE2 data acquisition sub-score, patient age, patient percent with high school diploma, patient income, patient region urban versus rural, patient comorbidity, patient prior hospitalization, patient breast biopsy or mammogram in previous year, and self referral for screening available. 499 patients had missing values for percent with a high school diploma and family income, and were not included in the regression model.

^b^in Canadian Dollars.

**Figure 1:**
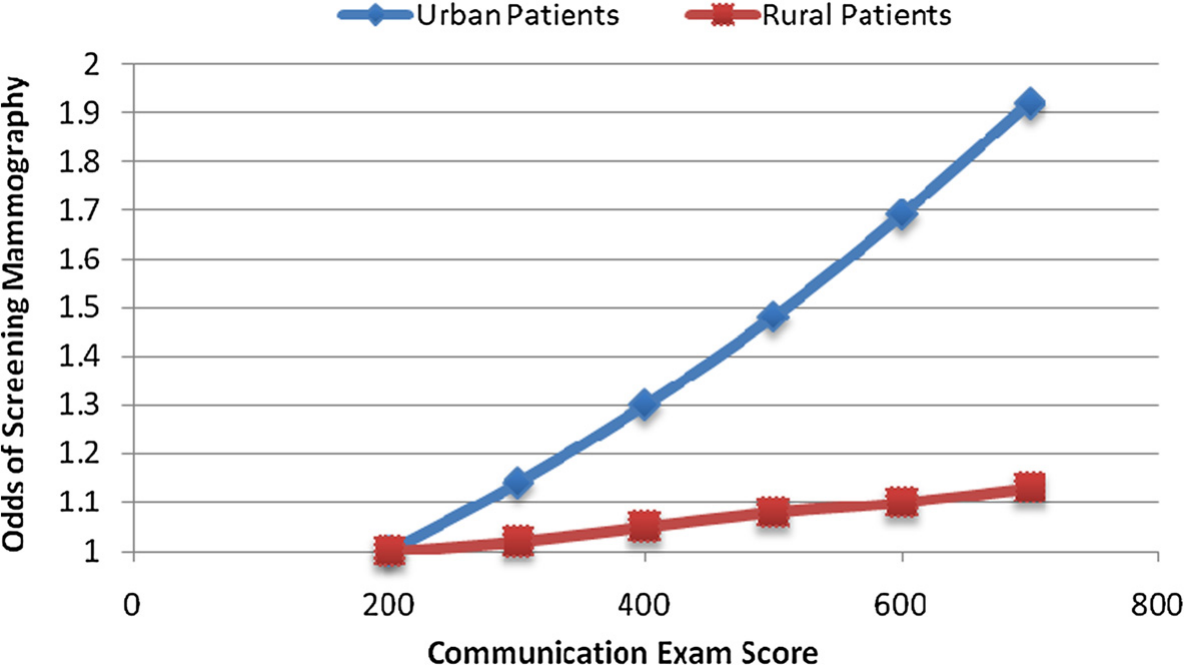
Modification of the effect of better communication abilities on the odds of mammography screening by urban versus rural patient residence.

The odds of ordering an SM were 20 % higher for female physicians relative to males (OR: 1.20, 95 % CI: 1.07, 1.35). In addition, odds of ordering a screening (and not diagnostic) mammogram were 59 % higher for surgeons compared to family/general physicians (OR: 1.59, 95 % CI: 1.22, 2.06) (Table 4). The odds of SM increased by 24 % for 2 standard deviations increase in communication score (OR: 1.24, 95 % CI: 1.11, 1.38). There was no significant relationship between data acquisition skills or overall clinical skills examination score and SM use. The association between communication and SM was not attenuated by practice type (surgical versus non-surgical) and experience (p-value for interaction: 0.75). However, a physician’s communication ability had a significantly stronger association with the likelihood of SM for urban compared to rural patients (interaction between communication score and patient’s place of residence: p-value <0.0001) (Figure 1). Among patients located in urban areas, a 2 SD increase in physician communication ability increased the odds of SM by 30 % (OR: 1.30, 95 % CI: 1.16, 1.46), whereas in rural areas the increase was only 6 % (OR: 1.06, 95 % CI: 0.80, 1.39).

**Table 4:** Physician characteristics and their relationship to providing mammography screening in the 12 months following a patient’s visit

	**Mammography screening**			
**Physician Characteristics**	**Yes**	**No**	**Odds Ratio**^**a**^**(95% CI)**	**P- Value**
	**N=32 689**	**N=64 019**		
**Demographics**	**No. (%)**	**No. (%)**		
**Sex**				
Male	11 107 (34.0)	25 366 (39.6)	reference	
Female	21 582 (66.0)	38 653 (60.4)	1.20 (1.07-1.35)	0.002
**Medical School**				
Canadian	29 297 (89.6)	56 626 (88.4)	reference	
International graduate	3 392 (10.4)	7393 (11.6)	1.12 (0.91-1.38)	0.30
**Specialty**				
Family medicine/general practice	29 640 (90.7)	59 54 (93.0)	reference	
Medical specialty	259 (0.8)	515 (0.8)	0.79 (0.53-1.18)	0.26
Surgical specialty	2 780 (8.5)	3 938 (6.2)	1.59 (1.22-2.06)	0.001
**Practice Experience**	**mean (SD)**	**mean (SD)**		
**Number of years of practice**, per year increase	3.4 (2.6)	3.7 (2.7)	0.96 (0.95-0.97)	<0.001
**Medical Knowledge & Clinical Decision Making Ability**	**mean (SD)**	**mean (SD)**		
**MCCQE1 score**, per 2 SD increase	512.3 (76.6)	510.7 (76.0)	1.05 (0.97-1.12)	0.22
**Communication Ability**	**mean (SD)**	**mean (SD)**		
**MCCQE2 Communication sub-score**, per 2 SD increase	516.4 (93.9)	507.6 (98.4)	1.24 (1.11-1.38)	<0.001
**MCCQE2 Data acquisition sub-score**, per 2 SD increase	531.0 (95.0)	531.1 (91.2)	0.98 (0.92-1.05)	0.58

^a^Estimated using the generalized estimating equation (GEE) extension of multivariate logistic regression, adjusting for physician sex, specialty, medical school within Canada, years of practice, MCCQE1 exam score, MCCQE2 communication sub-score, MCCQE2 data acquisition sub-score, patient age, patient percent with high school diploma, patient income, patient region urban versus rural, patient comorbidity, patient prior hospitalization, patient breast biopsy or mammogram in previous year, and self referral for screening available. 499 patients had missing values for percent with a high school diploma and family income, and were not included in the regression model.

## Discussion

This study demonstrates that communication skills may be an important predictor of SM use. Patients of physicians who are better communicators, as measured by a standardized national medical skills examination, are more likely to undergo screening mammograms.

Discussions in the medical setting significantly affect patient decisions and related health outcomes [16]. For over 50 % of women surveyed by Metsch, physicians represent the most important information source regarding breast health, surpassing telephone hotlines, family, friends and workplace [15]. We also know from other areas of prevention and health promotion (e.g. smoking cessation, weight loss) that a communication style based on clear information, emotional support, shared decision-making and agreement on the nature of the issue facilitates patient compliance [36,37]. The impact of physician communication is particularly important in breast cancer, where reduction of mortality is an attainable goal. Interestingly, many health care providers underestimate their patients’ information needs and desires [38,39]. Lack of clear physician recommendations, confusion about who is in charge of preventive care and lack of dialogue have been identified by women as important reasons not to undergo SM [11,16].

This study also confirmed that female doctors are more frequently successful at getting their patients to obtain SMs This has been noted by others as well and may reflect increased awareness of preventive measures and greater comfort discussing women’s health issues [31][5,11,40,41]. Interestingly, this difference between male and female physicians persisted even at equal communication skills scores. Cooper-Patrick hypothesizes that female physicians’ practice style may be more conducive to partnership-building and participatory decision-making [42]. Few communication assessment tools in medical education currently focus on the impact of gender. According to a recent systematic review by Dielissen et al, only 2 out of 21 communication skills assessment instruments explicitly presented gender as a criterion in their checklists [43].

Our results also showed that patients of surgical specialists have 60 % higher odds of obtaining an SM. Surgeons are also often the first ones consulted for breast masses and are heavily involved in all aspects of managing breast cancer, from work-up of mammographic abnormalities to anti-estrogen therapy.

The patient’s area of residence was another important predictor of SM use. Women living in rural areas had a higher chance of undergoing mammograms than their urban counterparts. This effect persisted despite a clustering of physicians from the bottom quartile of communication skills score into rural areas. A study of 3100 Ontario physicians noted that physicians working in urban practices were less likely to adhere to breast cancer screening recommendations [5]. Nutting hypothesizes that city physicians, in addition to having higher patient volume, are pressured by fee-for-service reimbursement, leaving little time for preventative measures [44]. Patient mobility is another factor that may influence use of SM in urban women. Physicians in rural areas are often fewer in number, integrated into the communities where they practice, and known to most, facilitating doctor-patient relationships and patient retention. In contrast, urban patients are mobile and have access to a multitude of physicians. This lack of continuity in primary care does not facilitate prevention strategies. Thus the stronger association between physician communication skills and mammography uptake for urban patients that we noted may reflect the importance of better communication in motivating women to overcome potential barriers to mammography that are more prevalent in urban areas (poverty, transportation barriers, unfavorable attitudes toward preventative care, mistrust of the medical system).

Consistent with previous studies, other patient-related factors impacting the use of SM included higher age, lower socioeconomic status and education [5,7,45,46]. In addition, women with a Charlson co-morbidity index ≥3 had 28 % less chance of undergoing SM compared to their healthy counterparts. Sicker patients may have more pressing health challenges. Furthermore, multiple medical co-morbidities may negate the long-term survival benefit of breast cancer screening.

Our study had several limitations. We measured communication skills using a validated physician skills evaluation exam applied at the end of training shown to predict a wide array of outcomes [28-30]. Nevertheless, these scores do not provide the accuracy that more logistically involved approaches offer (e.g. direct observation of clinical encounters). The poor-to-moderate reliability of the MCCQE2 communication score component probably led to underestimating the strength of relationship between SM use and physician communication skills [47]. If we adjust for attenuations produced by unreliability, the true estimate of the association would increase from an OR of 1.24 to an OR between 1.34 (α = 0.5) and 1.51 (α = 0.5). We were unable to include information on patient/physician preferred language. Linguistic proficiency is shown to affect patient decisions regarding treatment and providing informed consent [48,49]. It can also be argued that women with a greater overall interest in health and preventative care may preferentially seek health care providers with a specific practice style, including superior communication skills [50]. Finally, we were not able take into account patients’ place of birth and, if applicable, immigration details, even though country of origin and length of time since immigration may influence SM use [51,52].

## Conclusions

Doctor-patient communication involves integrating complex data and sharing of information in a collaborative fashion [53]. These skills are not developed through experience alone; they can be taught and assessed with evaluation [17,20]. This the first study confirming that standardized assessment of physician communication skills at the end of training is associated with outcomes in breast cancer screening during subsequent practice. In addition, we showed that end-of-training standardized test results for these physicianship-related skills are more strongly associated with future use of life-saving screening tests than test results for doctors’ knowledge of preventive medicine. Future research should examine whether early interventions to enhance competence in communication skills for physicians with lower scores can increase their ability to provide patients with screening procedures.

## Abbreviations

SM, screening mammography; MCC, Medical Council of Canada; MCCQE2, Medical Council of Canada – clinical skills examination; MCCQE1, Medical Council of Canada – knowledge and decision-making examination; RAMQ, Régie de l’assurance-maladie du Québec; ICD, International Classification of Diseases; GEE, Generalized Estimating Equation; SD, Standard Deviation.

## Competing interests

The authors declare that they have no competing interests.

## Authors’ contributions

ANM data analysis, manuscript drafting, critical review, DD manuscript drafting, critical review, ND study design, data analysis, critical review, TE study design, data analysis, critical review, KR study design, data analysis, critical review, AJ manuscript drafting, critical review, SM manuscript drafting, critical review, DB manuscript drafting, critical review, MA study design, data analysis, critical review and RT study design, data analysis, manuscript drafting, critical review. All authors read and approved the final manuscript.

## Pre-publication history

The pre-publication history for this paper can be accessed here:

http://www.biomedcentral.com/1472-6963/12/219/prepub

## References

[B1] Humphrey LL Helfand M Chan BK Woolf SH Breast cancer screening: a summary of the evidence for the U.S. Preventive Services Task Force Ann Intern Med 2002 137 347 360 12204020 10.7326/0003-4819-137-5_part_1-200209030-00012

[B2] Calonge N Calonge N Petitti DB Screening for breast cancer: U.S. Preventive Services Task Force recommendation statement Ann Intern Med 2009 151 716 726 W-236 19920272 10.7326/0003-4819-151-10-200911170-00008

[B3] Tonelli M Gorber SC Joffres M Recommendations on screening for breast cancer in average-risk women aged 40–74 years CMAJ 2011 183 1991 2001 22106103 10.1503/cmaj.110334 PMC3225421

[B4] Preventive Services Task Force recommendation statement Ann Intern Med 2009 151 716 726 W-236 19920272 10.7326/0003-4819-151-10-200911170-00008

[B5] Abdel-Malek N Chiarelli AM Sloan M Stewart DE Mai V Howlett RI Influence of physician and patient characteristics on adherence to breast cancer screening recommendations Eur J Cancer Prev 2008 17 48 53 10.1097/CEJ.0b013e32809b4cef 18090910

[B6] Fenton JJ Franks P Reid RJ Elmore JG Baldwin LM Continuity of care and cancer screening among health plan enrollees Med Care 2008 46 58 62 10.1097/MLR.0b013e318148493a 18162856

[B7] Schueler KM Chu PW Smith-Bindman R Factors associated with mammography utilization: a systematic quantitative review of the literature J Womens Health (Larchmt) 2008 17 1477 1498 10.1089/jwh.2007.0603 18954237

[B8] Lewis BG Halm EA Marcus SM Korenstein D Federman AD Preventive services use among women seen by gynecologists, general medical physicians, or both Obstet Gynecol 2008 111 945 952 10.1097/AOG.0b013e318169ce3e 18378755

[B9] Swan J Breen N Coates RJ Rimer BK Lee NC Progress in cancer screening practices in the United States: results from the 2000 National Health Interview Survey Cancer 2003 97 1528 1540 10.1002/cncr.11208 12627518

[B10] Schoen RE Marcus M Braham RL Factors associated with the use of screening mammography in a primary care setting J Community Health 1994 19 239 252 10.1007/BF02260384 7929885

[B11] Van Harrison R Janz NK Wolfe RA Characteristics of primary care physicians and their practices associated with mammography rates for older women Cancer 2003 98 1811 1821 10.1002/cncr.11744 14584062

[B12] Lurie N Margolis KL McGovern PG Mink PJ Slater JS Why do patients of female physicians have higher rates of breast and cervical cancer screening? J Gen Intern Med 1997 12 34 43 9034944 10.1046/j.1525-1497.1997.12102.x PMC1497051

[B13] Holmboe ES Wang Y Meehan TP Association between maintenance of certification examination scores and quality of care for medicare beneficiaries Arch Intern Med 2008 168 1396 1403 10.1001/archinte.168.13.1396 18625919

[B14] Grady KE Lemkau JP McVay JM Reisine ST The importance of physician encouragement in breast cancer screening of older women Prev Med 1992 21 766 780 10.1016/0091-7435(92)90083-T 1438121

[B15] Metsch LR McCoy CB McCoy HV Pereyra M Trapido E Miles C The role of the physician as an information source on mammography Cancer Pract 1998 6 229 236 10.1046/j.1523-5394.1998.006004229.x 9767336

[B16] Royak-Schaler R Passmore SR Gadalla S Exploring patient-physician communication in breast cancer care for African American women following primary treatment Oncol Nurs Forum 2008 35 836 843 10.1188/08.ONF.836-843 18765331

[B17] Haq C Steele DJ Marchand L Seibert C Brody D Integrating the art and science of medical practice: innovations in teaching medical communication skills Fam Med 2004 36 Suppl S43 S50 14961402

[B18] Hulsman RL Ros WJ Winnubst JA Bensing JM Teaching clinically experienced physicians communication skills. A review of evaluation studies Med Educ 1999 33 655 668 10.1046/j.1365-2923.1999.00519.x 10476016

[B19] Epstein RM Franks P Fiscella K Measuring patient-centered communication in patient-physician consultations: theoretical and practical issues Soc Sci Med 2005 61 1516 1528 10.1016/j.socscimed.2005.02.001 16005784

[B20] Roter DL Stewart M Putnam SM Lipkin M Stiles W Inui TS Communication patterns of primary care physicians JAMA 1997 277 350 356 10.1001/jama.1997.03540280088045 9002500

[B21] Levinson W Roter DL Mullooly JP Dull VT Frankel RM Physician-patient communication. The relationship with malpractice claims among primary care physicians and surgeons. JAMA 1997 277 553 559 10.1001/jama.1997.03540310051034 9032162

[B22] Beckman HB Markakis KM Suchman AL Frankel RM The doctor-patient relationship and malpractice. Lessons from plaintiff depositions Arch Intern Med 1994 154 1365 1370 10.1001/archinte.1994.00420120093010 8002688

[B23] Taylor DM Wolfe R Cameron PA Complaints from emergency department patients largely result from treatment and communication problems Emerg Med (Fremantle) 2002 14 43 49 10.1046/j.1442-2026.2002.00284.x 11993834

[B24] Moore PJ Adler NE Robertson PA Medical malpractice: the effect of doctor-patient relations on medical patient perceptions and malpractice intentions West J Med 2000 173 244 250 10.1136/ewjm.173.4.244 11017984 PMC1071103

[B25] Mandin H Dauphinee WD Conceptual guidelines for developing and maintaining curriculum and examination objectives: the experience of the Medical Council of Canada Acad Med 2000 75 1031 1037 10.1097/00001888-200010000-00024 11031155

[B26] Page G Bordage G Allen T Developing key-feature problems and examinations to assess clinical decision-making skills Acad Med 1995 70 194 201 10.1097/00001888-199503000-00009 7873006

[B27] Reznick RK Blackmore D Dauphinee WD Rothman AI Smee S Large-scale high-stakes testing with an OSCE: report from the Medical Council of Canada Acad Med 1996 71 S19 S21 8546771 10.1097/00001888-199601000-00031

[B28] Cadieux G Abrahamowicz M Dauphinee D Tamblyn R Are physicians with better clinical skills on licensing examinations less likely to prescribe antibiotics for viral respiratory infections in ambulatory care settings? Med Care 2011 49 156 165 10.1097/MLR.0b013e3182028c1a 21206293

[B29] Tamblyn R Abrahamowicz M Dauphinee D Influence of physicians' management and communication ability on patients' persistence with antihypertensive medication Arch Intern Med 2010 170 1064 1072 10.1001/archinternmed.2010.167 20585073

[B30] Tamblyn R Abrahamowicz M Dauphinee D Physician scores on a national clinical skills examination as predictors of complaints to medical regulatory authorities JAMA 2007 298 993 1001 10.1001/jama.298.9.993 17785644

[B31] Haggerty J Tamblyn R Abrahamowicz M Beaulieu MD Kishchuk N Screening mammography referral rates for women ages 50 to 69 years by recently-licensed family physicians: physician and practice environment correlates Prev Med 1999 29 391 404 10.1006/pmed.1999.0558 10564631

[B32] Tamblyn R Abrahamowicz M Dauphinee WD Association between licensure examination scores and practice in primary care JAMA 2002 288 3019 3026 10.1001/jama.288.23.3019 12479767

[B33] Canada S Postal Code Conversion File (PCCF) Reference Guide 2007 Minister of Industry, Ottawa 45

[B34] Poirier A Programme quebecois de depistage du cancer du sein 2004 Governement of Quebec, sociaux Mdlseds, ed. quebec City 1998–2003

[B35] Theberge I Hebert-Croteau N Langlois A Major D Brisson J Volume of screening mammography and performance in the Quebec population-based Breast Cancer Screening Program CMAJ 2005 172 195 199 10.1503/cmaj.1040485 15655240 PMC543982

[B36] Rost KM Flavin KS Cole K McGill JB Change in metabolic control and functional status after hospitalization. Impact of patient activation intervention in diabetic patients Diabetes Care 1991 14 881 889 10.2337/diacare.14.10.881 1773686

[B37] Jones LW Courneya KS Fairey AS Mackey JR Effects of an oncologist's recommendation to exercise on self-reported exercise behavior in newly diagnosed breast cancer survivors: a single-blind, randomized controlled trial Ann Behav Med 2004 28 105 113 10.1207/s15324796abm2802_5 15454357

[B38] Goldberg R Guadagnoli E Silliman RA Glicksman A Cancer patients' concerns: congruence between patients and primary care physicians J Cancer Educ 1990 5 193 199 10.1080/08858199009528064 2261341

[B39] Suominen T Leino-Kilpi H Laippala P Who provides support and how? Breast cancer patients and nurses evaluate patient support Cancer Nurs 1995 18 278 285 7664255

[B40] Henderson JT Weisman CS Physician gender effects on preventive screening and counseling: an analysis of male and female patients' health care experiences Med Care 2001 39 1281 1292 10.1097/00005650-200112000-00004 11717570

[B41] Flocke SA Gilchrist V Physician and patient gender concordance and the delivery of comprehensive clinical preventive services Med Care 2005 43 486 492 10.1097/01.mlr.0000160418.72625.1c 15838414

[B42] Cooper-Patrick L Gallo JJ Gonzales JJ Race, gender, and partnership in the patient-physician relationship JAMA 1999 282 583 589 10.1001/jama.282.6.583 10450723

[B43] Dielissen P Bottema B Verdonk P Lagro-Janssen T Attention to gender in communication skills assessment instruments in medical education: a review Med Educ 2011 45 239 248 10.1111/j.1365-2923.2010.03876.x 21299599

[B44] Nutting PA Baier M Werner JJ Cutter G Conry C Stewart L Competing demands in the office visit: what influences mammography recommendations? J Am Board Fam Pract 2001 14 352 361 11572540

[B45] Rahman SM Dignan MB Shelton BJ Factors influencing adherence to guidelines for screening mammography among women aged 40 years and older Ethn Dis 2003 13 477 484 14632267 PMC2848385

[B46] Meissner HI Breen N Taubman ML Vernon SW Graubard BI Which women aren't getting mammograms and why? (United States) Cancer Causes Control 2007 18 61 70 10.1007/s10552-006-0078-7 17186422

[B47] Cleary T Linn RL Walster GW Effect of reliability and validity on power of statistical tests Sociol Methodol 1970 2 130 138

[B48] Cowan EA Calderon Y Gennis P Macklin R Ortiz C Wall SP Spanish and English video-assisted informed consent for intravenous contrast administration in the emergency department: a randomized controlled trial Ann Emerg Med 2007 49 30 30 e1-3 10.1016/j.annemergmed.2006.07.934 17011074

[B49] Schenker Y Wang F Selig SJ Ng R Fernandez A The impact of language barriers on documentation of informed consent at a hospital with on-site interpreter services J Gen Intern Med 2007 22 Suppl 2 294 299 17957414 10.1007/s11606-007-0359-1 PMC2078548

[B50] Franks P Jerant AF Fiscella K Shields CG Tancredi DJ Epstein RM Studying physician effects on patient outcomes: physician interactional style and performance on quality of care indicators Soc Sci Med 2006 62 422 432 10.1016/j.socscimed.2005.05.027 15993531

[B51] Ivanov LL Hu J Leak A Immigrant women's cancer screening behaviors J Community Health Nurs 2010 27 32 45 10.1080/07370010903466163 20131135

[B52] Echeverria SE Carrasquillo O The roles of citizenship status, acculturation, and health insurance in breast and cervical cancer screening among immigrant women Med Care 2006 44 788 792 10.1097/01.mlr.0000215863.24214.41 16862042

[B53] Epstein RM Hundert EM Defining and assessing professional competence JAMA 2002 287 226 235 10.1001/jama.287.2.226 11779266

